# Supplementary material for: Venomix: a simple bioinformatic pipeline for identifying and characterizing toxin gene candidates from transcriptomic data
Source: PeerJ. 2018 Jul 31;6:e5361. doi: 10.7717/peerj.5361 (PMC6074769; doi:10.7717/peerj.5361)
Supplement: Supplemental Information 3 [file peerj-06-5361-s003.gz › FinalOutput_E-6/Zinc_metalloproteinase-disintegrin-like_moojenin_1/finaltree.pdf]

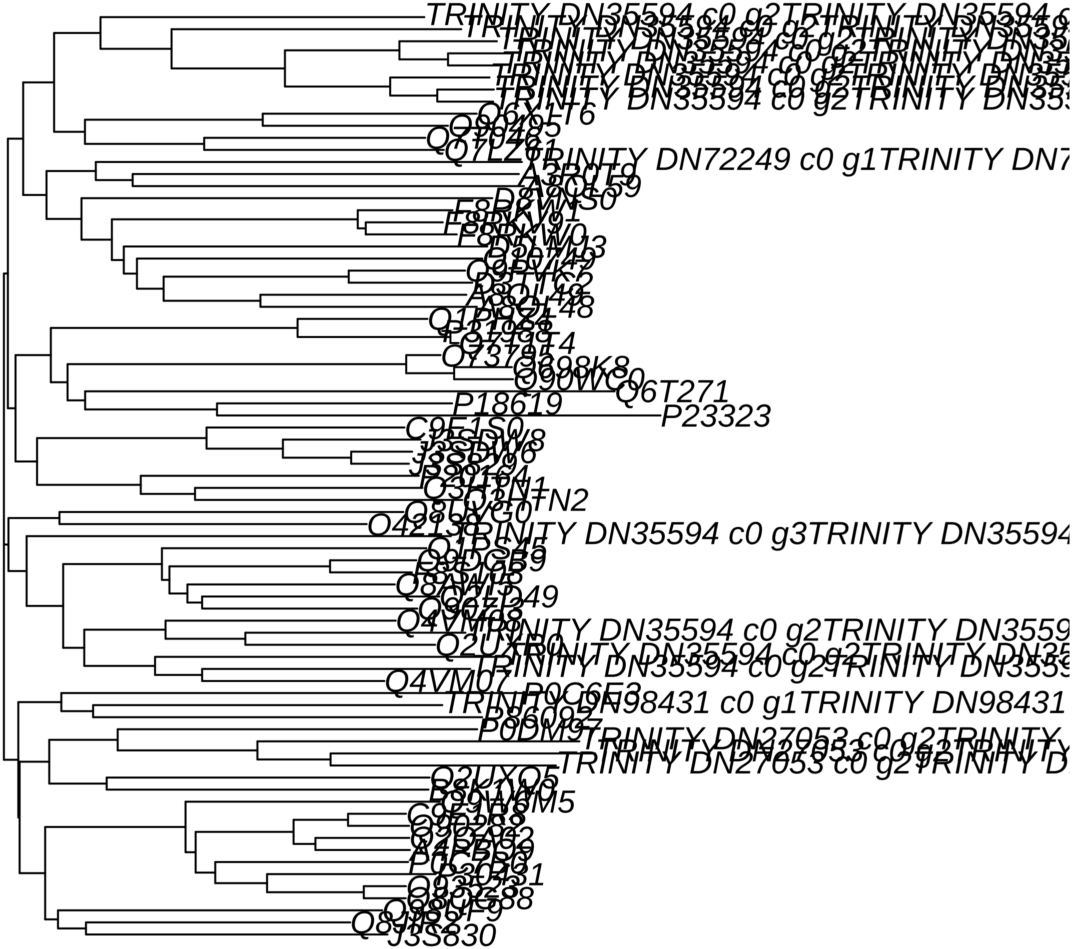

TRINITY DN35594 c0 g2TRINITY DN35594
TRINITY DN35594 c0 g2TRINITY DN35594 c0 g2TRINITY DN35594 c0 g2TRINITY DN35594 c0
Q6X1T6
Q90485
Q7T0461
Q7LZ61
TRINITY DN72249 c0 g1TRINITY DN7
A3R0T9
A2CL59
D2KWS0
F8RKV01
F8RKW0
F8RKW3
D2KX2
Q9PVJ2
D2DI49
Q9TWC2
A8QL48
Q1RP74
P21631
Q7T114
Q7T4Q5
Q98K8
Q90WC0
Q6T271
P18619
P23323
C9E1S0
J3SDW8
J3SDW6
J3SEZ4
P30021
Q3HTN1
Q3HTN2
Q8UVG0
O42139
TRINITY DN35594 c0 g3TRINITY DN35594
Q1RS45
Q8UCB9
P86155
Q8AWB3
Q6EZD9
Q4VM08
TRINITY DN35594 c0 g2TRINITY DN3559
Q2UXR0
TRINITY DN35594 c0 g2TRINITY DN3559
Q4VM07
P0C6E3
TRINITY DN98431 c0 g1TRINITY DN98431
P86092
P0DM9
TRINITY DN27053 c0 g2TRINITY
TRINITY DN27053 c0 g2TRINITY D
Q2UXQ5
B5KVW6
Q9W6M5
C9E1S8
Q9DG82
Q4RPB9
A1C2R0
P0C5P3
Q9DE88
Q8QFB
Q8JJ65
Q9PUF9
J3S830
